# Revision arthroplasty using a custom-made implant in the course of acetabular loosening of the J&J DePuy ASR replacement system - case report

**DOI:** 10.1097/MD.0000000000028475

**Published:** 2022-01-28

**Authors:** Aleksander Augustyn, Tomasz Stołtny, Dominika Rokicka, Marta Wróbel, Jan Pająk, Krystian Werner, Karol Ochocki, Krzysztof Strojek, Bogdan Koczy

**Affiliations:** a District Hospital of Orthopaedics and Trauma Surgery in Piekary Śląskie, Bytomska St. 62, Piekary Śląskie, Poland; b Department of Internal Diseases, Diabetology, and Cardiometabolic Diseases, School of Medicine with the Division of Dentistry in Zabrze, Medical University of Silesia in Katowice, Silesian Centre for Heart Diseases in Zabrze, M. Curie-Skłodowskiej St. 9, Zabrze, Poland.

**Keywords:** acetabular bone loss, case report, custom-made, J&J ASR, revision hip arthroplasty

## Abstract

**Rationale:**

Resurfacing arthroplasty using the J&J DePuy ASR system was withdrawn from surgical treatment due to the necessity of frequent revision procedures after its application. There have been many studies concerning treatment of acetabular bone loss using different operating techniques. However, we felt that data of custom - made implant usage in such cases is highly insufficient, and there is lack of evidence on its application in treatment of loosening of the previous implant. The aim of the study was to evaluate the effectiveness of surgical treatment with a custom-made implant in a patient with extensive acetabular bone loss after aseptic loosening of the acetabular component of the J&J DePuy ASR surface prosthesis in the early period of observation.

**Patient concerns:**

A 74-year-old patient was taken to the Orthopaedic Trauma Emergency Room due to increasing pain in the right hip for about 3 months. Nine years earlier he underwent resurfacing arthroplasty of the right hip using the J&J DePuy ASR method.

**Diagnoses:**

The imaging diagnostics (X-ray, computed tomography, ultrasound) revealed the presence of a pseudotumor and lysis around the acetabular implant, which caused a fracture in the acetabulum.

**Interventions:**

Revision arthroplasty of the right hip joint was performed with the removal of the ASR implant. During the procedure extensive bone defects were visualized, preventing the insertion of the revision acetabulum. After extensive plasticization of the defects with the use of allogeneic cancellous chips the “hanging hip” was left with the intention of making another attempt to insert the implant after the reconstitution of the acetabular bone. A computed tomography examination 2.5 years after the ASR removal revealed the lack of an adequate degree of bone remodeling for the planned implant. Arthroplasty using custom - made aMace Acetabular Revision System by Materialize was performed 3 years after the removal of ASR.

**Outcomes:**

Optimal implant adherence to the bone base and full osseointegration with the pelvic bone bearing has been achieved. Significant improvement in clinical parameters has been noted, with no complications in the postoperative period.

**Lessons:**

The use of an individual custom-made implant in extensive acetabular bone loss after aseptic loosening of the acetabular component of the J&J DePuy ASR surface prosthesis in patients is an effective method of surgical treatment.

## 1 Introduction

Resurfacing hip arthroplasty accounted for only 3.5% of all hip arthroplasty performed in 2020. Its indications include advanced degenerative changes, developmental dysplasia and bone necrosis within the hip joint.^[1]^

Data from the Australian Orthopaedic Society from 2007 and the National Joint Registry of England and Wales records from 2009 showed an alarmingly high percentage of J&J DePuy ASR revisions.^[2]^

Facing the challenge of revision hip arthroplasty a wide range of implant types is available which, if adequately selected and implanted, allows patients to return to satisfactory mobility. Custom-made implant systems meet the most difficult cases of extensive defects of the acetabular bone during revision arthroplasty.^[3]^

## 2 Case presentation

### 2.1 Patient presentation

A 74-year-old patient was taken to the Orthopaedic Trauma Emergency Room due to increasing pain in the right hip for about 3 months. Patient did not associate the occurrence of pain with the injury. He complained of continuous pain that intensified when the limb was loaded while walking, and that its mobility was restricted. The painkillers and anti-inflammatory drugs from the nonsteroidal anti-inflammatory drugs group, including nimesulide and tramadol, as well as well-balanced lifestyle, gave a poor feeling of relief. In the interview, the patient informed that he had undergone resurfacing of the right hip 9 years earlier using the J&J DePuy ASR method in metal-on-metal bearing.

### 2.2 Orthopedic examination and diagnosis

Initial orthopedic examination revealed inefficient gait with significant limitation on the right leg. The patient only moved with 2 elbow crutches. It was found that the operated limb was shortened by about 2 cm and the range of motion was limited by pain, including flexion up to 90°, and the internal and external rotation of the right hip was painfully abolished. The Anvil and Trendelenburg/Duchenne tests were negative.

**Figure 1:**
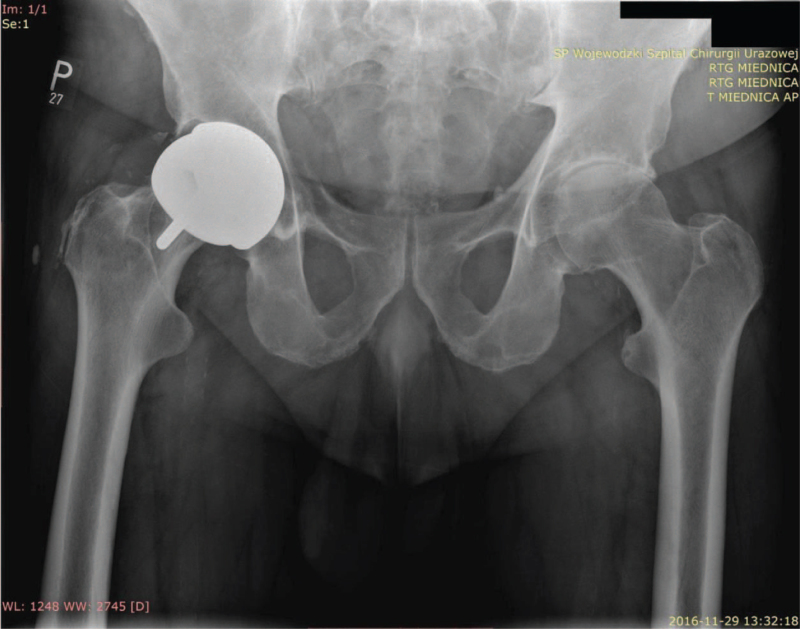
X-ray examination of the pelvis with hip joints in the AP projection. Visible lysis changes around the acetabular component of the right hip joint at the time of admission to the Orthopaedic Trauma Emergency Room, degenerative changes in the left hip joint.

Based on the obtained X-rays in classic projections for the evaluation of the hip replacement, the presence of a pseudotumor and lysis around the J&J DePuy ASR acetabular implant was suspected, which led to a fracture in the acetabulum. It was decided to extend the diagnostics to include ultrasound and CT, which confirmed the initial diagnosis, clarifying the nature of the bone defect and lysis in the pubic and hip bones. The dimensions of the pseudotumor located on the side of the lesser pelvis were determined to be 54 × 45 × 24 mm and the presence of a fluid reservoir within the joint was demonstrated in both studies (Fig. 1).

### 2.3 Treatment

After medical preparation, the patient was qualified for revision arthroplasty of the right hip joint with complete removal of the ASR implant and admitted to the department within 3 months of diagnosis. During the procedure, a pseudocapsule abundantly infiltrated with metallosis was found - a dark graphite color reaction. After removing the aseptically loosened acetabulum with the use of appropriate instruments, extensive bone defects were found within all walls and the bottom of the acetabulum bone, corresponding to grade IIIb on the Paprosky scale, as well as additional metallosis foci in the soft tissues of the minor pelvis.^[4]^

**Figure 2:**
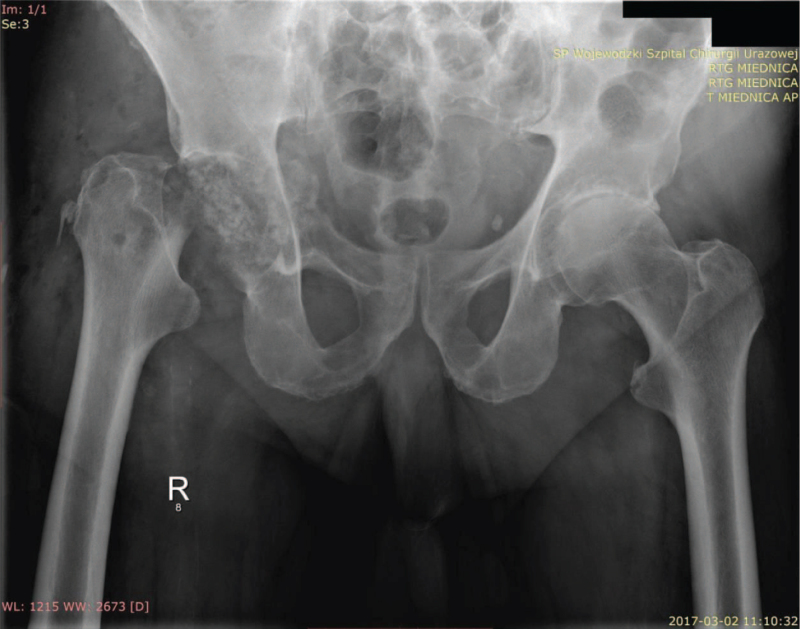
X-ray examination of the pelvis with hip joints. Condition after removal of the ASR implant, leaving the so-called “hanging joint” with visible extensive plastic surgery with allogeneic cancellous bone of the bottom of the acetabulum of the right hip joint, degenerative changes in the left hip.

The extent of bone defects made it impossible to reseat the revision acetabulum, both cementless and cemented, at the same time. A decision was made to perform extensive plastic surgery with allogeneic cancellous bone from the Tissue Bank in a volume of 120 cm^3^ with Stimulan.^[5]^ The head-cap of the J&J DePuy ASR System was resected to prepare the proximal end of the femur for the future placement of a classic cementless stem (Fig. 2).

**Figure 3:**
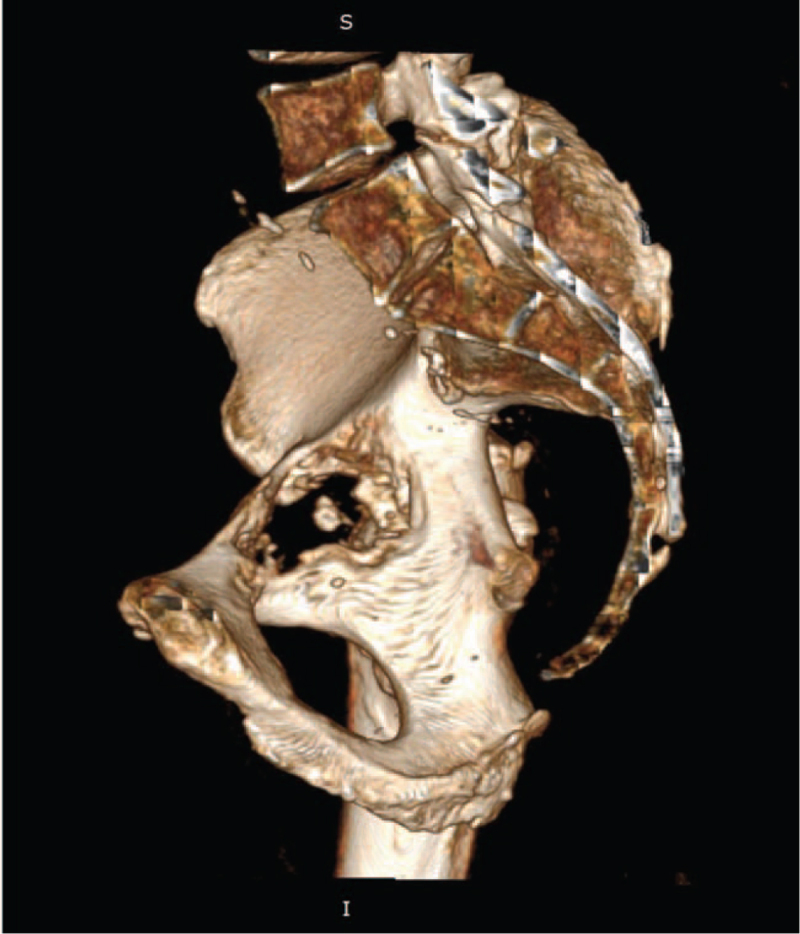
Computed tomography of the right hip joint in 3D imaging, view from the side of the smaller pelvis. Extensive bone loss in the bottom of the acetabulum and quadrilateral plate, after 2.5 yrs of bone remodelling, corresponding to grade IIIA on the Paprosky scale.^[4]^

The patient was prepared for reoperation during a series of visits to the Trauma and Orthopaedic Clinic, awaiting bone reconstruction within the acetabulum and creating conditions for the implantation of the revision acetabulum. A control computed tomography (CT) scan of the pelvis was performed every 6 months to assess the reconstruction within the defect after cancellous bone allografting. In the meantime, the patient learned to walk with 2 and then with 1 crutch. A CT scan 2.5 years after the ASR removal surgery revealed an insufficient degree of bone remodelling for the placement of a cementless revision cup, therefore it was decided to qualify the patient for an individual custom-made implant (Fig. 3).

Finally, 3 years after the removal of the ASR implant, the patient was admitted to the ward for a revision operation using a custom-made implant by Materialize aMace Acetabular Revision System distributed in Poland by Massmedica S.A., made of a titanium-aluminium-vanadium alloy.^[6]^ The physical examination revealed a shortening of the right lower limb by approx. 6 cm. During the 5-and-a-half-hour surgical procedure, placing the patient on his side (Hardinge access), using the acetabular gauge and the pelvic gauge in 3D technology, resection of the pubic, ischial, and iliac bones was performed and the acetabulum was refreshed with milling cutters according to the manufacturer's scheme. Optimal adhesion of the implant to the bone substrate was obtained, and then it was stabilized with 9 corkscrews, achieving a result consistent with preoperative planning. At a later stage, the Zimmer Muller cup was placed on the bone cement and the classic J&J DePuy Corail No. 15 stem with a collar was implanted.

### 2.4 Outcomes after surgery

A control X-ray after the procedure confirmed the images obtained intraoperatively - the correct seating of the prosthesis components in the anatomical position and full coverage of bone defects. The patient underwent initial rehabilitation in the ward, including the load on the operated limb. He was discharged home 12 days after the surgery. The patient was moving under the protection of 2 elbow crutches with the contact of the operated limb with the ground without actively transferring the load. The wound was healing by primary adhesion (Fig. 4 A and B).

**Figure 4:**
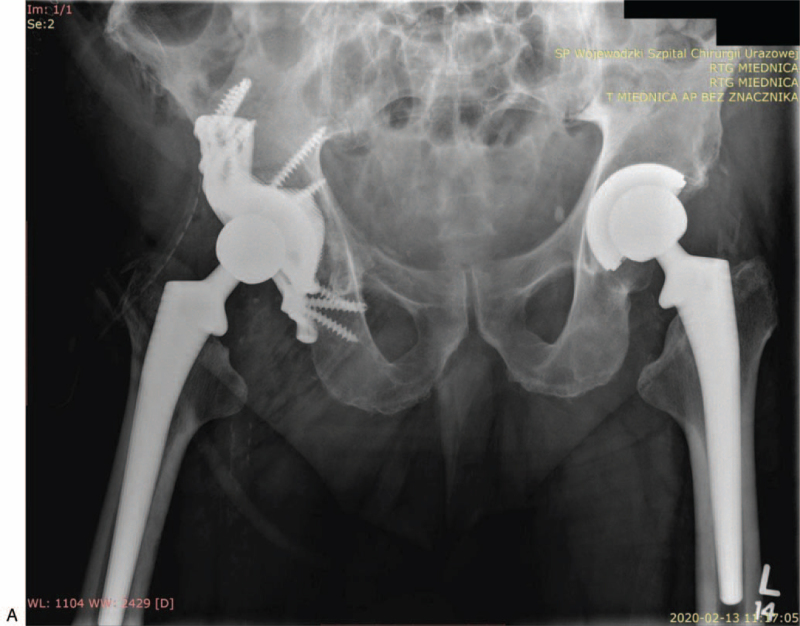
(A) X-ray of pelvis with hip joints (AP view) after revision surgery with custom-made endoprosthesis and J&J DePuy Corail head and stem on the right side, condition after left hip arthroplasty using J&J Corail method. (B) Axial X-ray of the right hip joint after revision surgery using a custom-made implant and a J&J DePuy Corail head and stem.

**Figure 4 (Continued):**
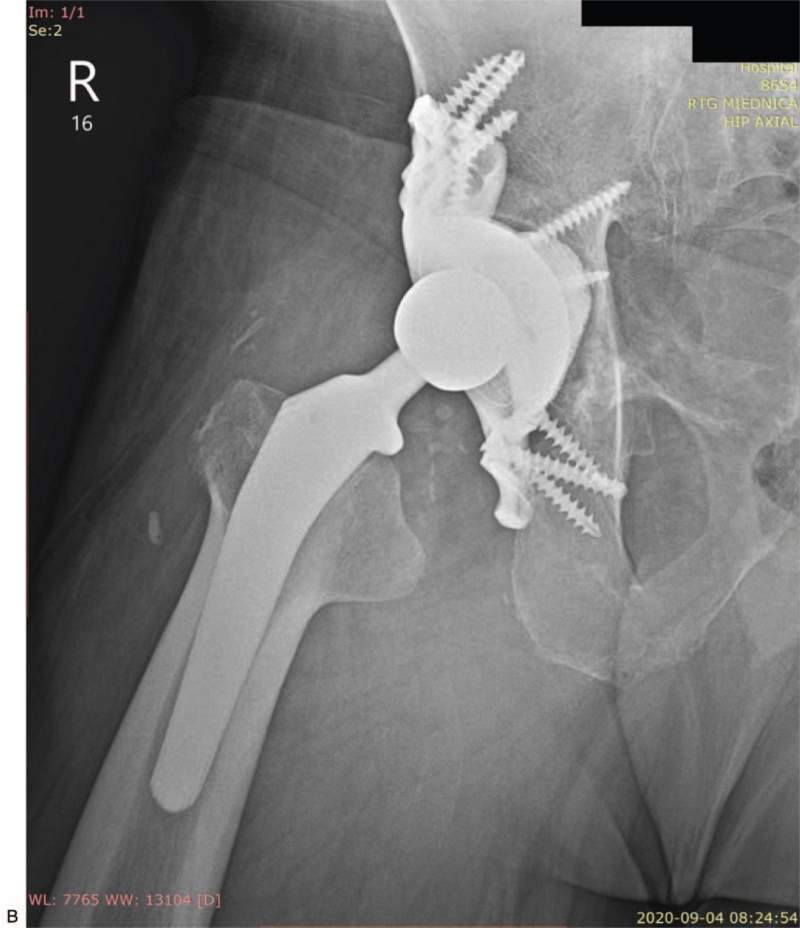
(A) X-ray of pelvis with hip joints (AP view) after revision surgery with custom-made endoprosthesis and J&J DePuy Corail head and stem on the right side, condition after left hip arthroplasty using J&J Corail method. (B) Axial X-ray of the right hip joint after revision surgery using a custom-made implant and a J&J DePuy Corail head and stem.

**Follow-up:** A detailed postoperative follow-up examination showed a significant improvement in clinical parameters compared to the state before the custom-made implant surgery (Tables 1 and 2).

**Table 1:** Clinical status before and after the operation using custom - made implant.

Scale/symptom	Before the operation	After the operation
HHS	20.95	80.85
WOMAC-HIP	38.30	82.00
Lovett scale	III	V
VAS	6	1
Trendelenburg/Duchenne	+++	+

HHS = Harris Hip score, VAS = visual analog scale, WOMAC = Western Ontario and McMaster Universities Arthritis Index.

**Table 2:** Right hip joint status before and after the operation using custom - made implant.

Mobility/activity	Before the operation	After the operation
Flexion	45°	95°
Internal rotation	0°	40°
External rotation	0°	45°
Adduction	10°	20°
Abduction	15°	50°
The length of the lower limbs	Lower right limb shortening 6 cm	No lower right limb shortening by 6 cm
Possible walking time	Walk 15 min, significant limp	Walk > 60 min, limp

## 3 Discussion

The goal of the creators of the J&J DePuy ASR system was to use it especially among young and active patients, whose life expectancy created a high probability of revision surgery in the future. The use of the implant introduced in 2003, thanks to resection of only the degenerated articular surface of the femoral head, was to minimize the loss of bone tissue in the proximal end of the femur.^[2]^

As a result of numerous reports in the literature that confirmed the unacceptably high frequency of revision procedures, in August 2010 the ASR implant was finally withdrawn from distribution by the manufacturer. Data from the National Joint Registry for England and Wales from 2010 indicate a 12% percentage of revision operations over a 5-year follow-up period.^[7]^

In an extensive study, Bozic et al^[8]^ identified the most common causes of acetabular revision: instability/dislocation (33.0%), mechanical loosening (24.3%), osteolysis around the implant (8.1%), abrasion of the bearing surface (8.0%), bacterial infection (4.7%), periprosthetic fracture (1.8%). In a patient treated in our centre, there was extensive lysis around the acetabular component of the surface prosthesis, which had an influence on periprosthetic fracture of the acetabulum of the pelvis bone.

Each complication of the acetabular component of the prosthesis may be accompanied by a defect in the bone tissue of one of the walls or the bottom of the acetabulum, which is assessed using multiple scales of radiological classification. In the case of hip surface arthroplasty, the surgical technique assumes first the implantation of the femoral component, the so-called “head cap” and the following specific adjustment to the size of the acetabulum. This procedure forced the operator to use a larger diameter of the socket milling cutters than it would have resulted from the size of the bone socket. Crawford et al^[9]^ demonstrated more than 3 times greater amount of removed acetabular bone tissue in the case of surface arthroplasty compared to classic arthroplasty with only twice less bone loss in the proximal femur.

At the same time, due to the inclination of the acetabulum deviating from the range of 45° ± 10° and the anteversion of 20° ± 10°, complications in the form of pseudotumor occur 4 times more often. The formation of a pseudotumor also occurs as a result of an inflammatory reaction to the presence of metal particles in periprosthetic soft tissues, usually in a metal-on-metal bearing.^[10]^ The acetabular inclination of the ASR prosthesis in the described patient after primary surgery was 65°. When reporting to the Orthopaedic Trauma Emergency Room, 9 years after the endoprosthesis was placed and it was amounted to 68°.

Custom-made triflange acetabular component elements are implants adapted to the patient, created as a result of detailed preoperative planning. Three-dimensional CT scan is used to create an acrylic model of the affected pelvis. This model includes the planned hip rotation centre and determines the location and length of the screws that will be used to fix it. After the model structure is agreed between the manufacturer and the surgeon, a custom, porous or hydroxyapatite-coated titanium implant with 3 “flanges” is created. Custom-made triflange acetabular component is able to cover acetabular bone defects in a more rigid and stable manner and can therefore be used to achieve bone remodelling even in the absence of continuity of the acetabular ring.^[11]^

In their study, Castagnini et al^[12]^ performed isolated acetabular revision surgeries of J&J DePuy ASR prostheses in 18 patients with various degrees of the extent of the acetabular bone loss due to the presence of the above-mentioned implant. In addition, each patient had periprosthetic metallosis. The method used by them was the installation of a high-porosity, titanium revision cup with a ceramic insert, supported in some cases with bone allografts or screws. They obtained satisfactory results in terms of pain reduction in the period of over 5 years follow-up (Harris Hip score [HHS] 88.3 ± 9.2; preoperative - 50.3 ± 4.6). Additionally, a significant reduction in the concentration of Cr and Co ions was observed in patients undergoing revision surgery.

Fröschen et al^[13]^ in a study involving 68 patients after revision hip arthroplasty with a high degree of Paprosky IIIa/b acetabular bone defect, in whom custom-made implants were used, obtained satisfactory results of clinical and radiological improvement - HHS 61.1 points out of 21 points preoperatively; accompanied, however, by a disturbingly high number of periprosthetic infections of 22%.

The original idea for the treatment of our patient, which was abandoned, was the use of a revision cup using bone allografts from the Tissue Bank. Kostensalo et al^[14]^ analyzed in their work the use of the above-mentioned implants. They found 73% survival of the implants used in patients with extensive bone tissue loss of the acetabulum in the mean follow-up period of 7 years. Unfortunately, more than 80% of 16 patients requiring revision had again aseptic loosening of the acetabulum.

Therefore, based on the literature data, we finally used a custom-made system, obtaining satisfactory clinical (HHS, Western Ontario and McMaster Universities Arthritis Index [WOMAC]-Hip, visual analog scale) and radiological effects during the observation period of 14 months.

## 4 Conclusions

1. Revision arthroplasty with the use of a custom-made implant in the course of aseptic loosening of the acetabular component of the J&J DePuy ASR prosthesis in patients with extensive pelvic annulus defect IIIa acc. Paprosky is an effective method of surgical treatment during the observation period of 14 months
2. The use of a custom-made implant affects:
  - a significant reduction in pain
  - compensation of the shortening of the operated limb
  - improving the range of motion
  - and significantly reduces the static-dynamic failure occurring before the surgery in the Anvil and Trendelenburg tests, and improves the clinical condition in the following evaluation sheets - HHS 80.85, WOMAC HIP 82.00 compared to the state before surgery - HHS 20.95, WOMAC HIP 38.30.

## Author contributions

AA wrote the draft of the manuscript and participated in the follow-up examination of the patient and clinical material. TS coordinated and helped to draft and finalize the manuscript. DR and MW participated in the writing of the draft of the manuscript. JP, KW, and KO performed the concluding surgery and followed up the patient. KS and BK have been involved in drafting the manuscript and revising it critically. All authors read and approved the final manuscript.

**Conceptualization:** Krzysztof Strojek.

**Data curation:** Aleksander Augustyn.

**Formal analysis:** Tomasz Stołtny, Dominika Rokicka, Marta Wróbel, Krzysztof Strojek.

**Investigation:** Jan Pająk, Karol Ochocki.

**Methodology:** Aleksander Augustyn, Tomasz Stołtny.

**Project administration:** Bogdan Koczy.

**Resources:** Dominika Rokicka, Marta Wróbel, Jan Pająk, Krystian Werner, Karol Ochocki.

**Software:** Aleksander Augustyn.

**Supervision:** Tomasz Stołtny.

**Validation:** Tomasz Stołtny, Krystian Werner.

**Writing – original draft:** Aleksander Augustyn.

**Writing – review & editing:** Aleksander Augustyn.
